# A large COVID-19 outbreak in a high school 10 days after schools’ reopening, Israel, May 2020

**DOI:** 10.2807/1560-7917.ES.2020.25.29.2001352

**Published:** 2020-07-23

**Authors:** Chen Stein-Zamir, Nitza Abramson, Hanna Shoob, Erez Libal, Menachem Bitan, Tanya Cardash, Refael Cayam, Ian Miskin

**Affiliations:** 1 Jerusalem District Health Office, Ministry of Health, Jerusalem, Israel; 2 The Hebrew University of Jerusalem, Faculty of Medicine, Braun School of Public and Community Medicine, Jerusalem, Israel; 3 Clalit health services, Jerusalem District, Jerusalem, Israel; 4 Meuchedet health services, Jerusalem District, Jerusalem, Israel; 5 Maccabi Healthcare services, Jerusalem and Shfela Region, Israel; 6 Leumit Health Services, Jerusalem District, Jerusalem, Israel

**Keywords:** COVID-19, Outbreak, High School, Children and adolescents

## Abstract

On 13 March 2020, Israel’s government declared closure of all schools. Schools fully reopened on 17 May 2020. Ten days later, a major outbreak of coronavirus disease (COVID-19) occurred in a high school. The first case was registered on 26 May, the second on 27 May. They were not epidemiologically linked. Testing of the complete school community revealed 153 students (attack rate: 13.2%) and 25 staff members (attack rate: 16.6%) who were COVID-19 positive.

As part of the coronavirus disease (COVID-19) pandemic containment measures, Israel's government declared complete closure of all educational facilities on 13 March 2020. Limited schools reopening (kindergartens, grades 1–3 and 11–12) only in small groups was approved on 3 May 2020. Subsequently, all school classes reopened on 17 May 2020, with requirement for daily health reports, hygiene, facemasks, social distancing and minimal interaction between classes. Ten days later, the first major COVID-19 school outbreak in Israel emerged in a high school. The first case was registered on 26 May and the second on 27 May. The two cases were not epidemiologically linked. Testing of the complete school community revealed 153 students (attack rate: 13.2%) and 25 staff members (attack rate: 16.6%) who were COVID-19 positive. Overall, some 260 persons were infected (students, staff members, relatives and friends). In this report, we aim to describe the investigation and epidemiological characteristics of the school's outbreak.

## Outbreak description and epidemiological investigation

School 1 is a regional public school; students arrive from suburbs and neighbourhoods, by public or school bus. It contains 1,190 students aged 12–18 years (grades 7–12) and 162 staff members. The school reopened after 2 months’ closure on Monday, 18 May 2020. Students returned to their previous classrooms and received instructions on preventive procedures. On 19–21 May (Tuesday to Thursday), an extreme heatwave occurred. Hence, the Ministry of Health exempted schoolchildren from facemasks for these 3 days.

The first COVID-19 case (Student A) was notified on 26 May 2020. The source of infection was unknown. Close contacts from household (n = 4), students (n = 50) and teachers (n = 14) were instructed to self-isolate. The second case (Student B) was notified on 27 May 2020. According to the epidemiological investigation, both students attended school during the days of 19–21 May and reported mild symptoms (anosmia, ageusia, fever and headache). They were from different grades and were not epidemiologically linked. 

With the emergence of two unrelated cases within 2 days, the district health office declared an ‘outbreak status' including school closure, isolation instructions and testing of the school community. During that long weekend (a Jewish holiday, 28–30 May 2020), mass COVID-19 testing was conducted as a joint effort of the school leadership and community, the four Health Funds, Magen David Adom (national emergency services organisation), the local municipality and the district health office.

Ten teachers and 26 students who had not attended school since reopening were excluded. Most of the remaining school community was tested, 151 of 152 staff members and 1,161 of 1,164 students. Overall, 153 students and 25 staff members were confirmed as COVID-19-positive. The data from the epidemiological investigation are shown in the Table. The COVID-19 rates differed between groups. Male cases were slightly overrepresented. The rate of cases reporting symptoms, upon meticulous questioning, was 43% (66/153) among students and 76% (19/25) among staff. The leading symptoms reported were cough, headache, fever, sore throat and myalgia. One emergency room visit was recorded and no hospitalisations.

**Table:** Epidemiological investigation data, COVID-19 outbreak, Israel, May 2020 (n = 1,316^a^)

Group	Number of persons	Number tested	Males		Confirmed cases		Males, of confirmed cases		Median age in years (cases)	Symptoms	
			n	%	n	Rate (%)	n	%		n	%
7th grade	197	197	106	53.8	40	20.3	25	62.5	13	19	47.5
8th grade	197	197	102	51.8	34	17.3	19	55.9	14	15	44.1
9th grade	187	187	94	50.3	61	32.6	32	52.5	15	30	49.2
10th grade	200	200	110	55.0	9	4.5	6	66.7	16	2	22.2
11th grade	195	194	98	50.5	6	3.1	3	50.0	17	0	0
12th grade	188	186	87	46.8	3	1.6	1	33.3	18	0	0
All students	1,164	1,161	597	51.4	153	13.2	86	56.2	15	66	43.1
Staff	152	151	51	33.8	25	16.6	9	36.0	40	19	76

COVID-19: coronavirus disease.

^a^ Overall 1,312 members of the school community were tested: 1,161 students and 151 staff.

**Figure 1:**
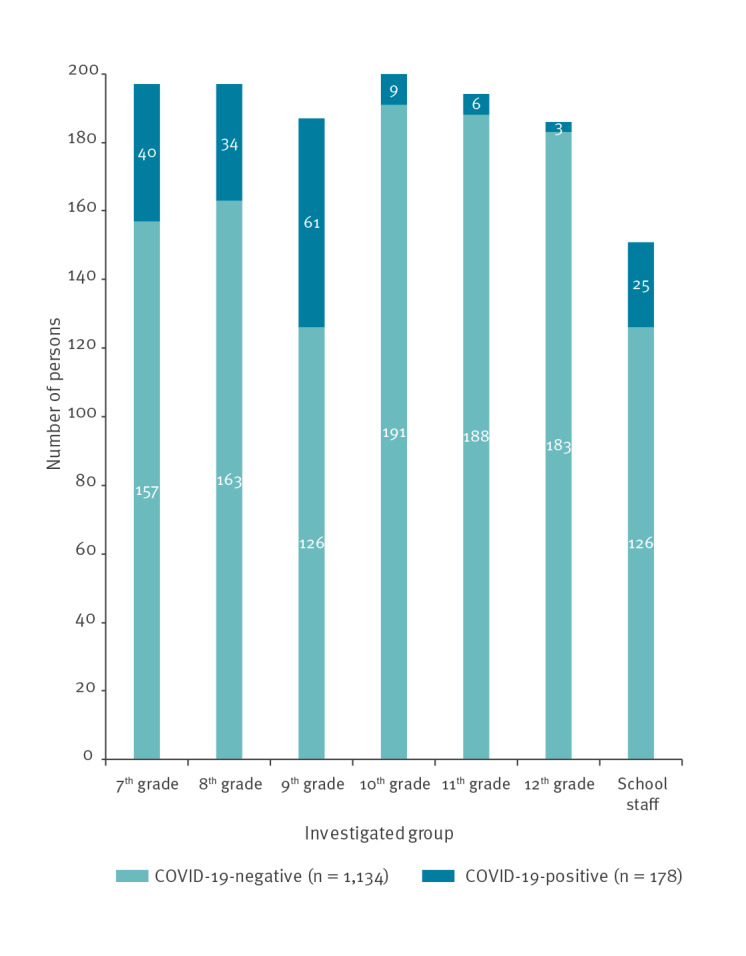
Results of COVID-19 testing, school outbreak, Jerusalem, May 2020 (n = 1,312)

COVID-19 rates were higher in junior grades (7–9) than in high grades (10–12) (Figure 1). The peak rates were observed in the 9th grade (20 cases in one class and 13 cases in two other classes) and the 7th grade (14 cases in one class). Of the cases in teachers, four taught all these four classes, two taught three of the four classes and one taught two of these four classes.

COVID-19: coronavirus disease.

An environmental school inspection reported crowded classes: 35–38 students per class, class area 39–49 m^2^, allowing 1.1–1.3 m^2^ per student (below the 1.5 m^2^ standard). Distancing among students and between students and teachers was not possible. Furthermore, during the extreme heatwave, air-conditioning functioned continuously in all classes. The air-conditioning system was separate for each class. The junior grades (7–9) and the high grades (10–12) are situated in one large building, yet in separate wings, and share the schoolyard and public spaces. According to the school schedule, students study 6 days (Sunday to Friday) for 38–40 h weekly (6.3–6.7 h daily on average). Daily travel time to school depends on distance and traffic conditions and lasts 20–45 min. Most students also participate in extracurricular activities such as sports teams or dance classes for an average of 2–4 h per week.

As at 30 June 2020, 100 of 153 (65.4%) students and 16 of 25 (64%) staff members have recovered (with two negative PCR results). Evaluating the recovery period revealed that 60% of asymptomatic cases recovered within 25 days vs only 37% of symptomatic cases.

## Cases outside the first affected school

By mid-June 2020, 87 additional confirmed COVID-19 cases had occurred among close contacts of the first school’s cases. These included siblings attending other schools, friends and participants in sports and dancing afternoon classes, students’ parents and family members of school staff. 

## COVID-19 cases age distribution in the Jerusalem district

**Figure 2:**
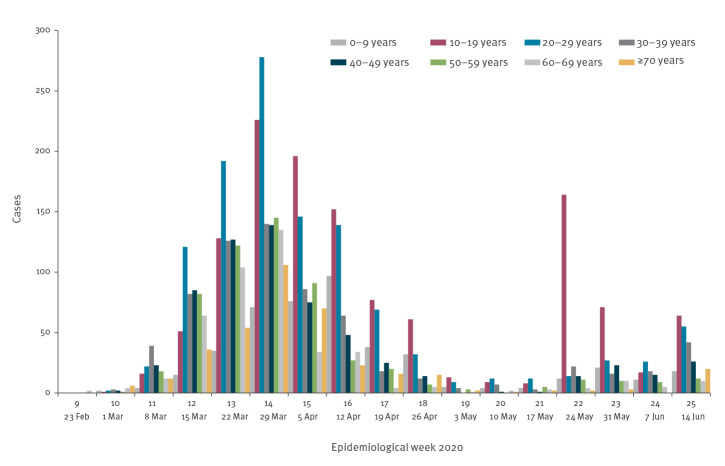
COVID-19 cases, Jerusalem, February–June 2020 (n = 5,519)

The large school outbreak led us to evaluate the age distribution of COVID-19 cases before and after schools' reopening. From week 9 to week 25 in 2020, 5,519 confirmed COVID-19 cases were reported in the Jerusalem district. As schools reopened on 17 May 2020, the evaluation point selected was 1 week later, on 24 May 2020 (week 22). The evaluation showed that before 24 May 2020, the proportion of the 10–19 years-olds (representing schoolchildren), was 19.8% (938/4,747) of cases in weeks 9–21, increasing to 40.9% (316/772) after 24 May 2020, in weeks 22–25 (Figure 2).

COVID-19: coronavirus disease.

**Figure 3:**
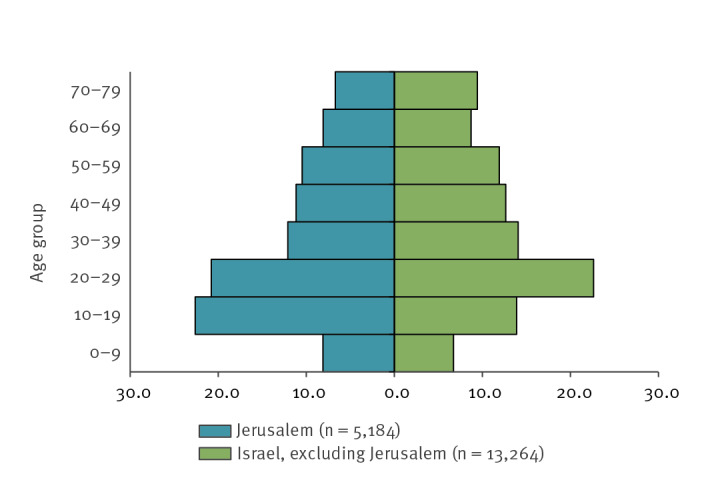
Age distribution of COVID-19 cases, Israel, May 2020 (n = 18,448)

From week 9 to week 24 in 2020, 18,448 confirmed COVID-19 cases were reported nationally, 5,184 cases in the Jerusalem district and 13,264 cases in all the other districts in Israel, excluding Jerusalem. The age pyramid of confirmed COVID-19 cases in the Jerusalem district vs nationally (excluding Jerusalem) showed a prominence of the 10–19 years-olds in Jerusalem, 22.6% vs. 13.9% in all the other districts (Figure 3). 

COVID-19: coronavirus disease.

## Discussion

On 27 January 2020, Israel's health minister declared COVID-19 infection a notifiable disease requiring immediate reporting. By 21 June 2020, some 20,778 confirmed COVID-19 cases had been reported with 306 fatalities [1]. Israel's population is 9.1 million (median age: 30 years) [2]. Like other countries, Israel implemented diverse containment measures including quarantine. Nationally, there are 1.7 million schoolchildren, 830,000 kindergarten children and 170,000 teachers and staff [3]. Full closure of educational facilities occurred on 13 March 2020. Elsewhere, 107 countries had implemented national school closures by 18 March 2020 [4].

COVID-19 cases are defined clinically (fever > 38 °C, cough, respiratory illness etc.) and epidemiologically. Laboratory confirmation requires detection of SARS-CoV-2 nucleic acid by PCR in nasopharyngeal swabs. The district health offices perform epidemiological investigations and contact tracing and issue isolation instructions and guidance to healthcare, educational and other facilities. The Health Funds, via community clinics, follow patients, refer to hospital if necessary and provide counselling to patients and families. The Jerusalem health office serves 1.25 million residents (median age: 23.5 years), characterised by moderate to low socioeconomic status and large households [5].

The high school outbreak in Jerusalem displayed mass COVID-19 transmission upon school reopening. The circumstances promoting infection spread involved return of teenage students to their regular classes after a 2-month closure (on 18 May) and an extreme heatwave (on 19 May) with temperatures rising to 40 °C and above [6] that involved exemption from facemasks and continuous air-conditioning. Classes in the first affected school had more than 30 students. Israel’s secondary school classes are crowded (average: 29 students in public schools) compared with the Organisation for Economic Cooperation and Development (OECD) average (23 students) [7]. COVID-19 in a school necessitates a prompt response. Classmates and teachers should be considered close contacts (particularly in crowded classes), as should students in groups mixing several classes, extra-curricular activities and school buses. Temporary school closure is prudent (especially in large regional schools) pending investigation results.

Most student cases presented with mild symptoms or were asymptomatic. Severe acute respiratory syndrome coronavirus 2 (SARS-CoV-2) infection in children and adolescents is considered mild compared with adults. A review of 18 studies (1,065 hospitalised paediatric patients) presented overall good prognosis for that age group [8]. A Chinese study of 171 paediatric cases infected with SARS-CoV-2 reported main signs of fever, cough and pharyngitis, 16% were asymptomatic [9]. In a European multicentre study (582 children), COVID-19 was usually mild, a small fraction developed severe disease and mortality was rare [10]. In a study in New York State, Kawasaki-like disease and myocarditis have been linked to COVID-19 infection, with the condition termed multisystem inflammatory syndrome (MIS-C) in children [11]. French paediatric surveillance data also support linkage between SARS-CoV-2 infection and MIS-C [12].

The role of children and adolescents in COVID-19 spread is equivocal; epidemiological data imply insignificance of children in transmission [13]. School closure is a public health tool in influenza pandemic preparedness plans, based on high infectiousness and susceptibility in schoolchildren and high contact rates [14]. School reopening policy after the COVID-19 lockdown varies considerably between nations and therefore requires ongoing assessment [13].

## Conclusions and recommendations 

COVID-19 prevention in schools involves studying in small groups and minimising student mixing in activities and transportation. Teachers and parents should lead by wearing facemasks, hand hygiene, keeping physical distance etc. School attendance should be avoided at any sign of illness. Learning from home may also reduce the need for class attendance. Outdoors classes should also be considered. COVID-19 prevention encompasses avoiding the ‘three Cs’: closed spaces with poor ventilation, crowded places and close-contact settings [15]. The European Centre for Disease Prevention and Control’s report on air-conditioning and ventilation systems and COVID-19 recommends increasing air exchange rate and outdoor air use and decreasing air recirculation, aiming to reduce spread in indoor spaces [16]. Finally, appropriate planning of COVID-19 prevention for the next school year is essential.
